# Dynamic Changes of Amplitude of Low-Frequency Fluctuations in Patients With Diabetic Retinopathy

**DOI:** 10.3389/fneur.2021.611702

**Published:** 2021-02-11

**Authors:** Xin Huang, Zhi Wen, Chen-Xing Qi, Yan Tong, Yin Shen

**Affiliations:** ^1^Eye Center, Renmin Hospital of Wuhan University, Wuhan, China; ^2^Department of Ophthalmology, Jiangxi Provincial People's Hospital, Nanchang, China; ^3^Department of Radiology, Renmin Hospital of Wuhan University, Wuhan, China; ^4^Medical Research Institute, Wuhan University, Wuhan, China

**Keywords:** diabetic retinopathy, dynamic amplitude of low-frequency fluctuation, functional magnetic resonance imaging, network centrality, functional network

## Abstract

**Background:** Growing evidence demonstrate that diabetic retinopathy (DR) patients have a high risk of cognitive decline and exhibit abnormal brain activity. However, neuroimaging studies thus far have focused on static cerebral activity changes in DR patients. The characteristics of dynamic cerebral activity in patients with DR are poorly understood.

**Purpose:** The purpose of the study was to investigate the dynamic cerebral activity changes in patients with DR using the dynamic amplitude of low-frequency fluctuation (dALFF) method.

**Materials and methods:** Thirty-four DR patients (18 men and 16 women) and 38 healthy controls (HCs) (18 males and 20 females) closely matched in age, sex, and education were enrolled in this study. The dALFF method was used to investigate dynamic intrinsic brain activity differences between the DR and HC groups.

**Results:** Compared with HCs, DR patients exhibited increased dALFF variability in the right brainstem, left cerebellum_8, left cerebellum_9, and left parahippocampal gyrus. In contrast, DR patients exhibited decreased dALFF variability in the left middle occipital gyrus and right middle occipital gyrus.

**Conclusion:** Our study highlighted that DR patients showed abnormal variability of dALFF in the visual cortices, cerebellum, and parahippocampal gyrus. These findings suggest impaired visual and motor and memory function in DR individuals. Thus, abnormal dynamic spontaneous brain activity might be involved in the pathophysiology of DR.

## Introduction

Diabetic retinopathy (DR) is a serious diabetic retinal microvascular complication (1). The global prevalence of DR is reportedly 34.6% worldwide (2). There are several risk factors for the development of DR, including diabetes duration, hemoglobin A1c (HbA1c) level, and blood pressure (3). DR is mainly divided into non-proliferative retinopathy and proliferative retinopathy (4). The retinal vasculature shares similar anatomical, physiological, and embryological characteristics with cerebral vessels. There is growing evidence to support a link between DR and microvascular stroke (5, 6). Furthermore, DR is a potential independent risk factor for cognitive decline (7, 8). However, the etiology of this increased risk is unclear.

Recently, growing neuroimaging studies using the fMRI method have demonstrated that DR patients are associated with abnormal brain activity. Wang et al. (9) demonstrated that DR patients had widespread abnormal ALFF values in the occipital gyrus, cerebellar lobe, and parahippocampal gyrus, relative to healthy controls (HCs). Wang et al. (10) revealed that DR patients showed lower degree centrality in the right inferior temporal gyrus and left subcallosal gyrus regions, as well as higher degree centrality in the bilateral precuneus relative to health controls. Liao et al. (11) demonstrated that DR patients had increased regional homogeneity (ReHo) values in the bilateral posterior lobes of the cerebellum relative to HCs and decreased ReHo values in the right anterior cingulate gyrus, right cuneus, bilateral precuneus, and left-middle frontal gyrus relative to healthy controls. DR leads to local brain activity changes, as well as brain functional network dysfunction. Our previous study demonstrated that DR patients had abnormal function in the default-mode, visual, salience, and sensorimotor networks (12). Notably, van Duinkerken et al. (13) demonstrated widespread brain network dysfunction. However, existing studies have mainly focused on static cerebral activity changes in DR patients. There is increasing evidence of the dynamic nature of brain activity and connections (14, 15). Thus, we presume that dynamic brain activity analyses can be used to deepen our understanding of neural mechanism changes in DR patients.

Low-frequency oscillations (<0.08 Hz) of blood-oxygenation-level dependent (BOLD) signaling in the human brain are physiologically meaningful (16, 17). The human brain is a complex dynamic system capable of non-stationary neural activity and rapid changes in neural interaction. Dynamic characteristics of brain activity are reportedly associated with various physiological functions, such as consciousness (18) and cognition (19). The ALFF method is a reliable and sensitive fMRI technology quantifying local intrinsic brain activity (20) Combining the ALFF with sliding-window approaches, the dALFF method can be used to calculate the variance of ALFF. The dALFF method provides a new approach for the investigation of dynamic brain activity (21). Recently, dALFF analysis was successfully applied to assess the dynamic cerebral activity changes in patients with generalized tonic-clonic seizures (22), poststroke aphasia (23), and schizophrenia (24). Thus, we hypothesized that DR patients might have dynamic cerebral activity changes.

To address this issue, the purpose of this study was to determine whether altered dynamic spontaneous neural activity was present in DR patients, using ALFF with sliding-window approaches for assessment.

## Materials and Methods

### Participants

Thirty-four DR patients (18 males and 16 females) and 38 healthy controls (HCs) (18 males and 20 females) matched for age, sex, education participated in the study.

The diagnostic criteria of DR patients were: (1) fasting plasma glucose ≥7.0 mmol/L, random plasma glucose ≥11.1 mmol/L, or 2-h glucose ≥11.1 mmol/L; (2) the non-proliferative DR group showed microaneurysms, hard exudates, and retinal hemorrhages.

The exclusion criteria of DR individuals in the study were: (1) proliferative DR with retinal detachment; (2) vitreous hemorrhage;

All HCs met the following criteria: (1) fasting plasma glucose <7.0 mmol/L, random plasma glucose <11.1 mmol/L, and HbA1c <6.5%; (2) no ocular diseases; (3) binocular visual acuity ≥1.0.

### Ethical Statement

The research protocol followed the Declaration of Helsinki and was approved by the medical ethics committee of the Renmin Hospital of Wuhan University. All subjects provided written informed consent to participate in the study.

### MRI Parameters

MRI scanning was performed on a 3-tesl magnetic resonance scanner (Discovery MR 750W system; GE Healthcare, Milwaukee, WI, USA) with an eight-channel head coil. All subjects underwent MRI scanning (8 min) with eyes closed without falling asleep. A total of 240 functional images were obtained. Detailed scanning parameters are shown in Table 1.

**Table 1:** The details on scanning parameters.

**Three-dimensional brain volume imaging (3D-BRAVO) sequence**		**Gradient-echo-planar imaging sequence**	
Repetition time/echo time	8.5/3.3	Repetition time/echo time	2,000 ms/25 ms
Slice thickness	1.0 mm	Slice thickness	3.0 mm
Acquisition matrix	256 × 256	Gap	1.2 mm
Field of view	240 × 240 mm^2^	Acquisition matrix	64 × 64
Flip angle	12°	Flip angle	90°
		Field of view	240 × 240 mm^2^
		Voxel size	3.6 × 3.6 × 3.6 mm^3^

### fMRI Data Processing

All pre-processing was performed using the toolbox for Data Processing & Analysis of Brain Imaging (DPABI, http://www.rfmri.org/dpabi) (25), using the following steps: (1) the first 10 volumes were removed and slice timing effects were motion corrected. (2) Individual 3D-BRAVO images were registered to the mean fMRI data (26) Normalized data [in Montreal Neurological Institute (MNI) 152 space] were re-sliced at a resolution of 3 × 3 × 3 mm^3^. (3) Detrending; (4) linear regression analysis was used to regress out several covariates. (5) temporal band-pass was filtered (0.01–0.08 Hz). No Scrubbing regression was not performed (27).

### dALFF Variance Computing

**Figure 1:**
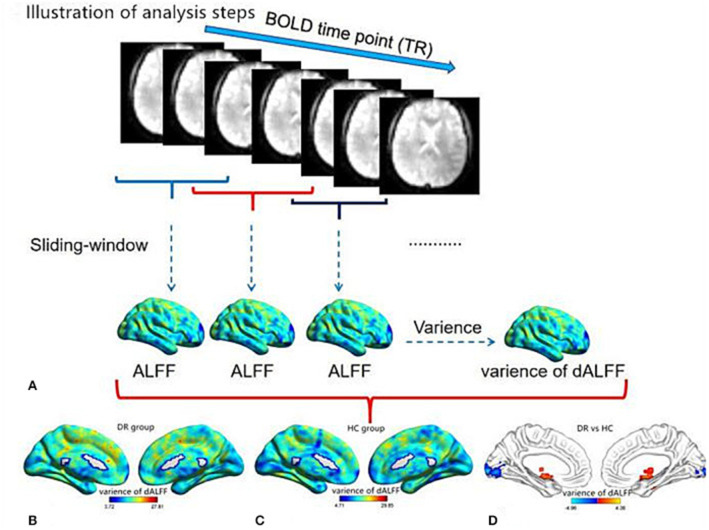
Illustration of analysis steps and temporal variability of dALFF pattern. **(A)** The pre-processed full-length BOLD fMRI time series was segmented into several sliding windows (50TRs). The temporal variability of the dALFF was defined as the variance of dALFF maps across the sliding windows. The pattern of temporal variability of the dALFF in the DR group **(B)** and HC group **(C)**. The different temporal variability of dALFF between two groups. **(D)** dALFF, dynamic amplitude of low-frequency fluctuation; DR, Diabetic Retinopathy; HC, Health Controls.

A sliding window approach was used to compute the dALFF using the Dynamic Brain Connectome (DynamicBC) toolbox (v2.0, www.restfmri.net/forum/DynamicBC) (28). For the sliding-window approach a window size of 50 TRs (100 s) and a window shifted by 10 TRs were selected (29) Figure 1.

### Clustering Analysis

A k-means clustering method was used to analyze dALFF of all subjects using the DynamicBC toolbox (v2.0, www.restfmri.net/forum/DynamicBC) (30). The Manhattan (L1) distance function method was performed to assess the reoccurrence over time in patterns of ALFF. The clustering centroids were used for the departure points to cluster all dALFF windows.

### Clinical Evaluation

The visual acuity of all subjects was measured by applying the logarithm of the minimum angle of the resolution table. All DR patients underwent biochemical examinations including for fasting blood glucose level, low density lipoprotein (LDL) cholesterol, high density lipoprotein (HDL) cholesterol, total cholesterol, and triglyceride.

### Statistical Analysis

The Chi-square (x2) test and independent-sample *t*-test were used to assess the clinical data between two groups using SPSS version 20.0.

A one-sample *t*-test was conducted to assess intra-group patterns of zdALFF maps and a two-sample *t*-test was used to compare zdALFF map differences between the two groups' regressed covariates of age and sex and FD. The Gaussian random field (GRF) method was used to correct for multiple comparisons (two-tailed, voxel-level *P* < 0.01, GRF correction, cluster-level *P* < 0.05).

Independent-sample *t*-test were performed to assess the different temporal properties of dALFF patterns between two groups including the mean dwell time (MDT) and the number of transitions (NT).

### Verification Analyses

To validate our dALFF findings, two different window lengths [30 TRs [60 s] and 100 TRs [200 s]] were calculated in the validation analysis.

## Results

### Demographic Measurements

There are no significant differences in age between the two groups. There are significant differences in BCVA (*p* < 0.001) between the two groups. Details are shown in Table 2.

**Table 2:** Demographics and visual measurements between two groups.

	**DR group**	**HC group**	***T*-values**	***P*-values**
Gender (male/female)	18/16	15/23	N/A	N/A
Age (years)	53.52 ± 8.67	47.10 ± 13.83	2.329	0.023
Handedness	34 R	38 R	N/A	N/A
BMI (kg/m^2^)	23.76 ± 2.33	23.02 ± 1.91	1.488	0.141
Duration of diabetes (years)	5.02 ± 6.67	N/A	N/A	N/A
BCVA-OD	0.48 ± 0.28	1.36 ± 0.15	−16.454	<0.001
BCVA-OS	0.41 ± 0.31	1.14 ± 0.20	−16.454	<0.001
HbA1c (%)	7.34 ± 1.34	N/A	N/A	N/A
Fasting blood glucose (mmol/L)	7.87 ± 2.54	N/A	N/A	N/A
LDL cholesterol (mmol/L)	2.22 ± 0.60	N/A	N/A	N/A
HDL cholesterol (mmol/L)	1.11 ± 0.28	N/A	N/A	N/A
Total cholesterol (mmol/L)	1.91 ± 1.38	N/A	N/A	N/A
Triglyceride (mmol/L)	3.69 ± 1.22	N/A	N/A	N/A
MoCA	24.97 ± 0.72	27.03 ± 0.82	−11.250	<0.001

*χ^2^-test for sex (n). Independent t-test for the other normally distributed continuous data (means ± SD). DR, diabetic retinopathy; HC, health control; N/A, not applicable; BCVA, best corrected visual acuity; OD, oculus dexter; OS, oculus sinister; Hb, glycosylated hemoglobin; BMI, body mass index; LDL, low density lipoprotein; HDL, high density lipoprotein*.

### Dynamic ALFF Variance Differences

**Figure 2:**
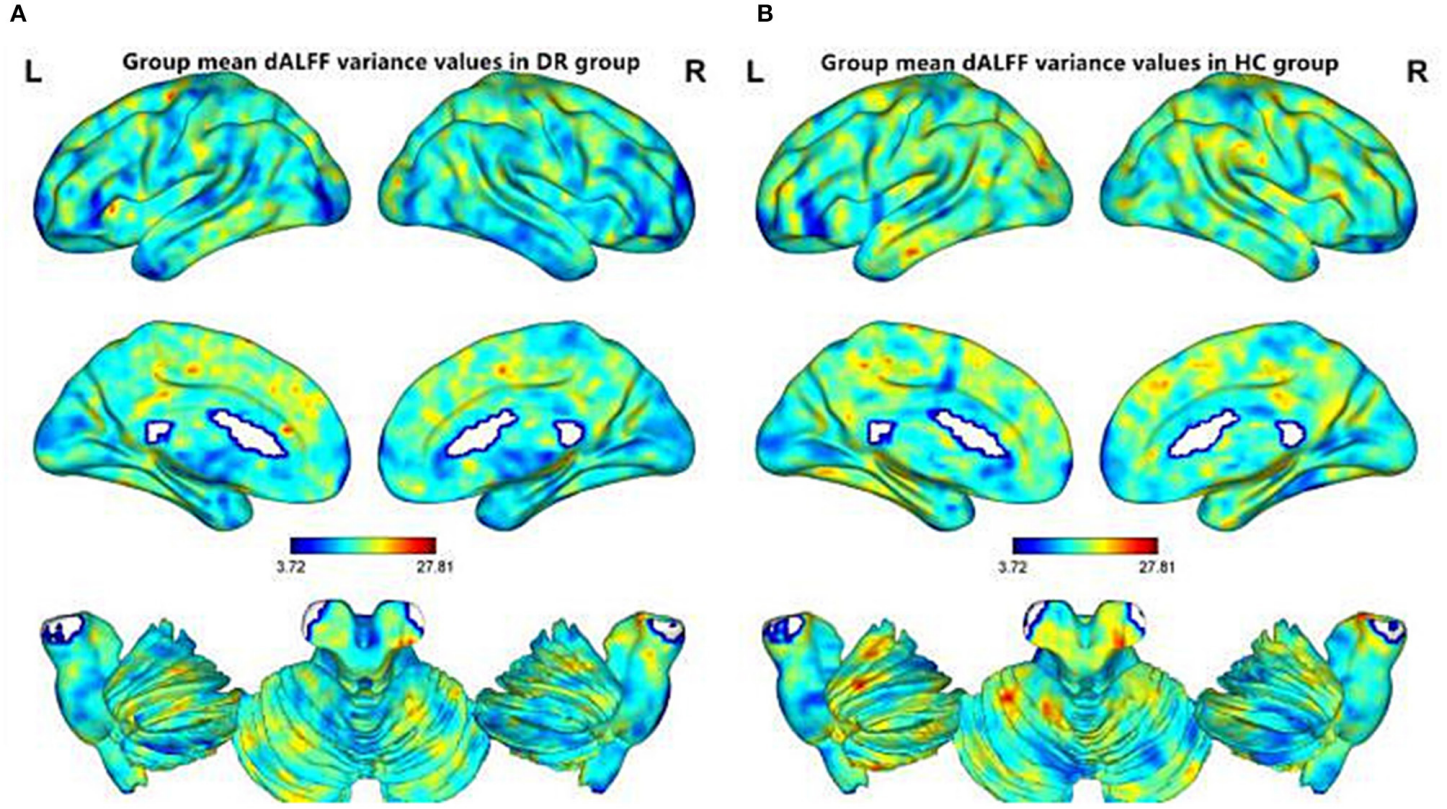
Spatial patterns of dALFF variance were observed at the group level in DR and HC groups. Within group mean dALFF variance maps within the DR **(A)** and HC **(B)**. dALFF, dynamic amplitude of low-frequency fluctuation; DR, Diabetic Retinopathy; HC, Health Controls; L, left; R, right.

**Figure 3:**
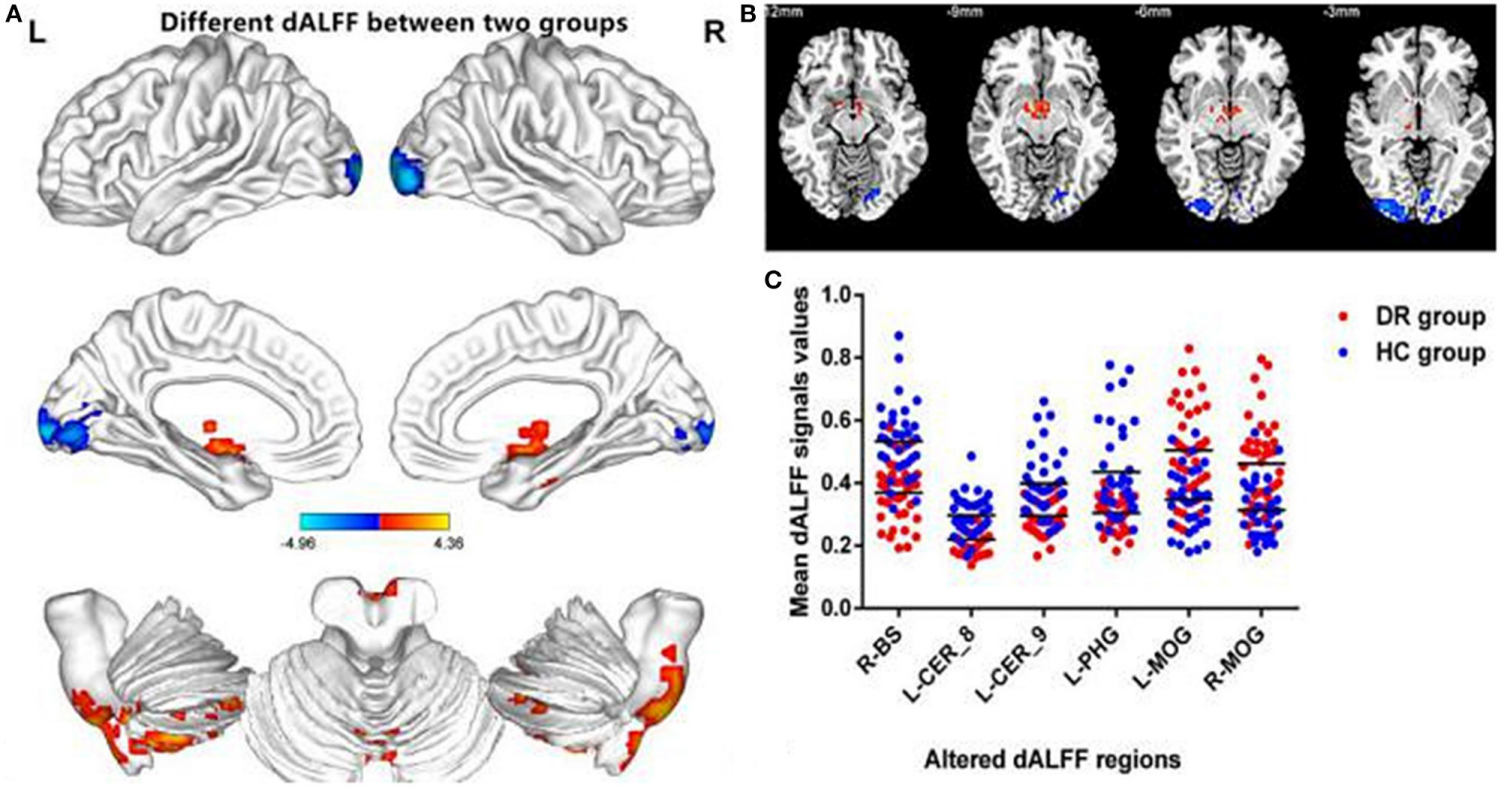
Comparison of different dALFF values between the DR group and HC group. Significant dALFF value differences were observed in the R-BS, L-CER_8, L-CER_9, L-PHG, L-MOG, R-MOG **(A,B)**. The mean values of altered dALFF values between the DR and HC groups **(C)**. dALFF, dynamic amplitude of low-frequency fluctuation; DR, Diabetic Retinopathy; HC, Health Controls; GRF, Gaussian random field; BS, Brainstem; CER, Cerebellum; PHG, Parahippocampal; MOG, middle occipital gyrus; L, left; R, right.

The spatial distribution of dALFF maps for the two groups is shown in Figure 2. Compared with HCs, DR patients exhibited increased dALFF variability in the right brainstem, left cerebelum_8, left Cerebelum_9, and left parahippocampal gyrus (Figures 3A,B (red) and Table 3). In contrast, DR patients exhibited decreased dALFF variability in the left middle occipital gyrus and right middle occipital gyrus (Figures 3A,B (blue) and Table 3). The mean values of altered dALFF values between the DR and HC groups (Figure 3C).

**Table 3:** Significant differences in the dALFF values between two groups.

**Condition/brain regions**		**BA**	**Peak T-scores**	**MNI coordinates**			**Cluster size (voxels)**
				**x**	**y**	**z**	
DR>HC	R-Brainstem	–	4.26	−9	−30	−48	239
DR>HC	L-Cerebelum_8	–	4.2701	−12	−66	−45	390
DR>HC	L-Cerebelum_9	–	3.5938	−6	−54	−54	36
DR>HC	L-Parahippocampal	34	3.8218	−15	0	−18	54
DR<HC	L-Middle Occipital gyrus	18	−4.9639	−24	−99	3	153
DR<HC	R-Middle Occipital gyrus	18	−4.2268	33	−93	0	196

*The statistical threshold was set at the voxel level with p < 0.01 for multiple comparisons using Gaussian random-field theory (two-tailed, voxel-level P < 0.01, GRF correction, cluster-level P < 0.05). dALFF, dynamic amplitude of low-frequency fluctuation; BA, Brodmann area; DR, diabetic retinopathy; HC, health control; MNI, Montreal Neurological Institute; GRF, Gaussian random field; L, left; R, right*.

### Clustered Dynamic ALFF States

**Figure 4:**
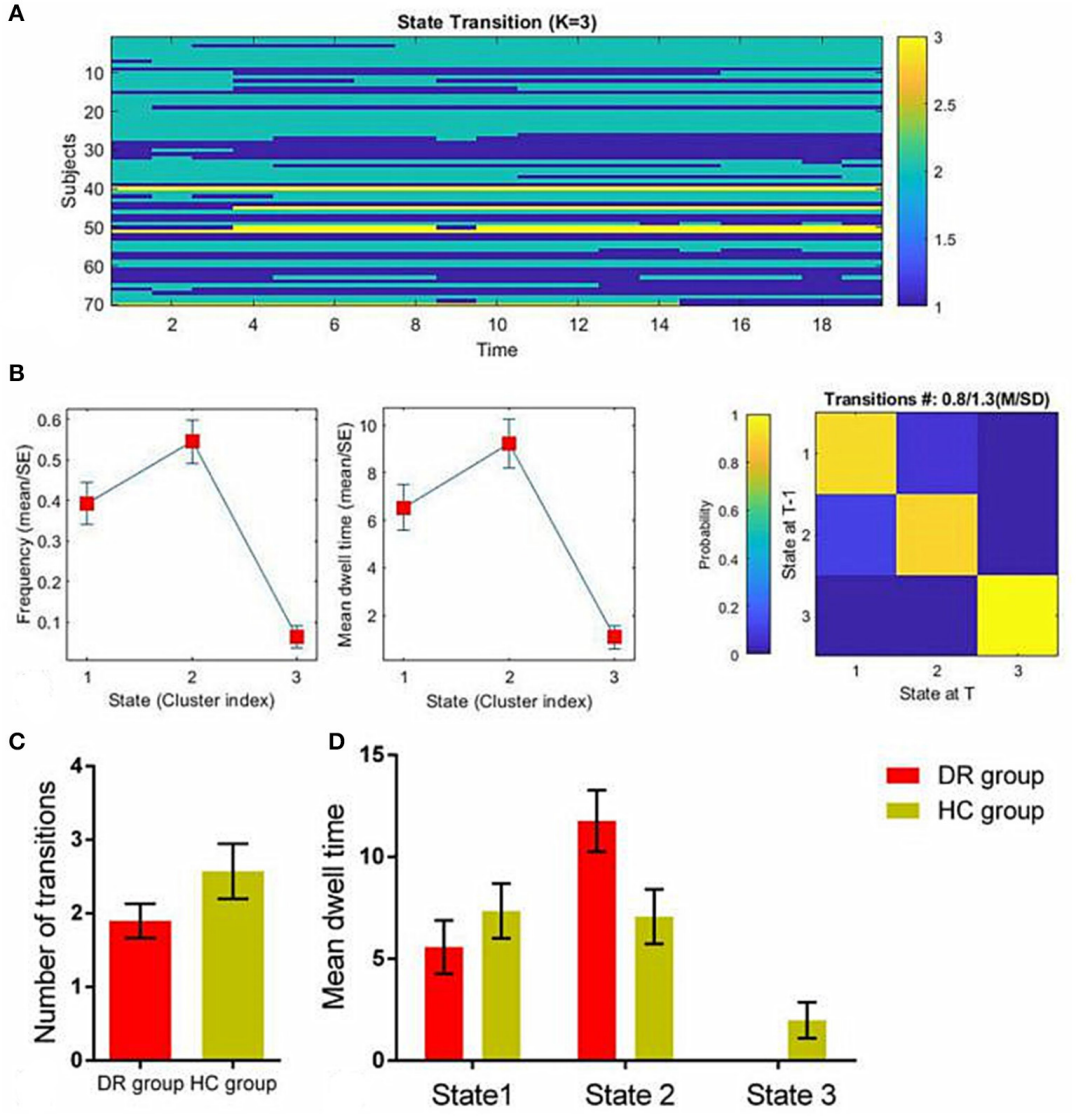
The temporal properties of dALFF patterns between the DR group and HC group. The K-means clustering method with three clusters **(A)**. Transition matrices of different states **(B)**. The number of transitions between states **(C)**. Mean dwell time **(D)**. dALFF, dynamic amplitude of low-frequency fluctuation; DR, Diabetic Retinopathy; HC, Health Controls.

All the subjects showed three different states (Figure 4A). The transition matrices of different states based on dALFF, were extracted using the K-means clustering method with three clusters (Figure 4B). Compared with the HC group, the DR group showed a shorter number of transitions between states (*t* = −1.524, *p* = 0.143) (Figure 4C). Moreover, the DR group showed a different mean dwell time in three states relative to the HC group (Figure 4D). The more details were showed in Table 4.

**Table 4:** The temporal properties of dALFF patterns between two groups.

	**DR group**	**HC group**	***T*-values**	***P*-values**
Number of transitions between states	1.90 ± 0.73	2.57 ± 1.40	−1.524	0.143
**Mean Dwell Time**				
State 1	5.58 ± 7.37	7.35 ± 8.29	−0.935	0.353
State 2	11.77 ± 8.51	7.07 ± 8.23	2.342	0.022
State 3	0.00 ± 0.00	1.98 ± 5.40	−2.267	0.029

*Independent t-test for the other normally distributed continuous data (means ± SD). DR, diabetic retinopathy; HC, health control*.

### Receiver Operating Characteristic Curve

**Figure 5:**
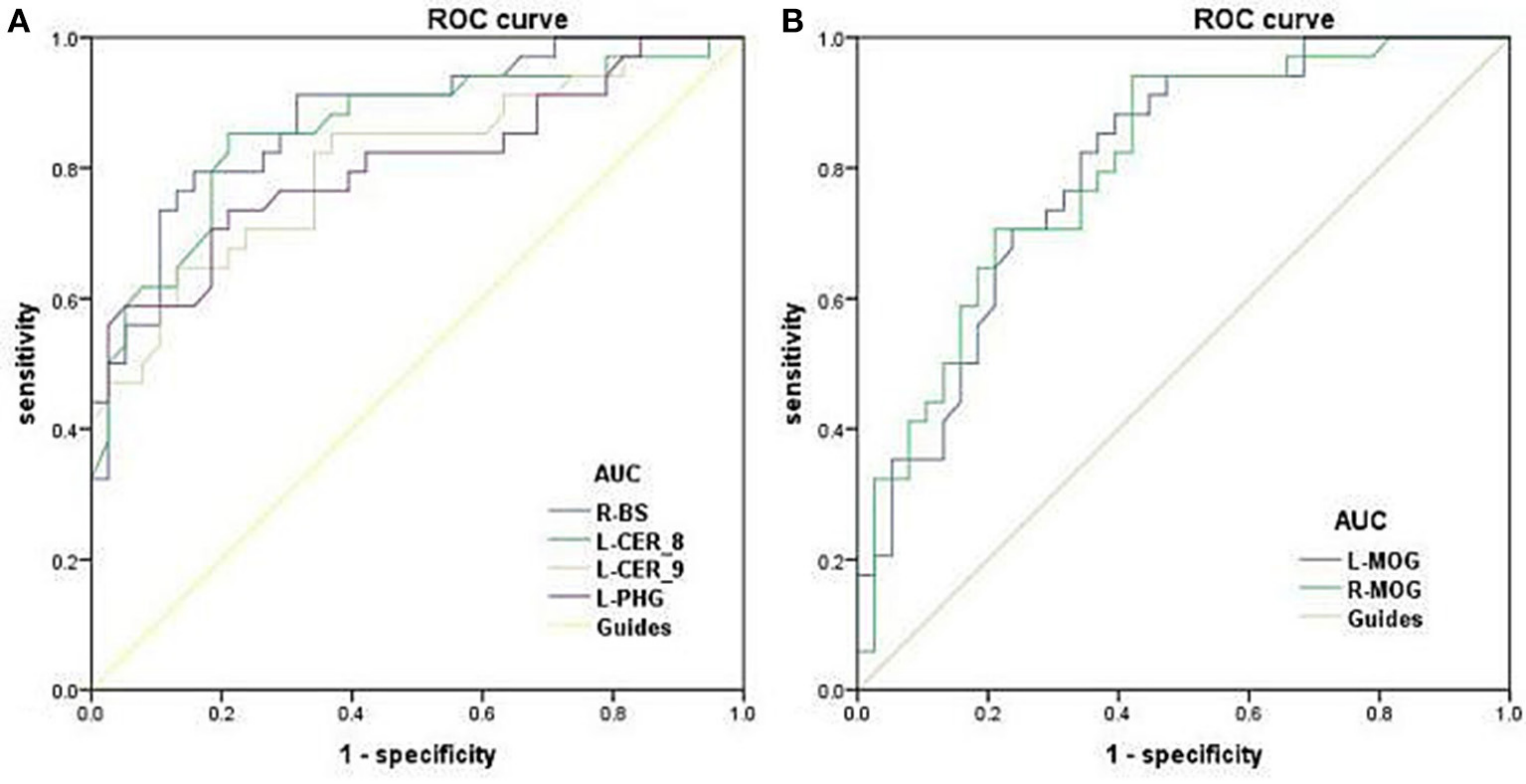
ROC curve analysis of the mean dALFF for altered brain regions. ROC curve in dALFF values: DR>HC, for R-BS 0.874 (*P* < 0.001; 95% CI: 0.794–0.955); for L-CER_8 0.859 (*P* < 0.001; 95% CI: 0.769–0.948); for L-CER_9 0.809 (*P* < 0.001; 95% CI: 0.708–0.910); for L-PHG 0.806 (*P* < 0.001; 95% CI: 0.701–0.910); **(A)** DR<HC, for L-MOG 0.800 (*P* < 0.001; 95% CI: 0.699–0.901); for R-MOG 0.803 (*P* < 0.001; 95% CI: 0.702–0.904); **(B)**. ROC, receiver operating characteristic; dALFF, dynamic amplitude of low-frequency fluctuation; AUC, area under the curve; BS, Brainstem; CER, Cerebelum; PHG, Parahippocampal; MOG, middle occipital gyrus; L, left; R, right.

The areas under the ROC curve for dALFF values were: DR>HC, for right brainstem 0.874 (*P* < 0.001; 95% CI: 0.794–0.955); for left cerebelum_8 0.859 (*P* < 0.001; 95% CI: 0.769–0.948); for left Cerebelum_9 0.809 (*P* < 0.001; 95% CI: 0.708–0.910); for left parahippocampal gyrus 0.806 (*P* < 0.001; 95% CI: 0.701–0.910) (Figure 5A); DR<HC, for left middle occipital gyrus 0.800 (*P* < 0.001; 95% CI: 0.699–0.901); for right middle occipital gyrus 0.803 (*P* < 0.001; 95% CI: 0.702–0.904); (Figure 5B).

### Verification Analyses

In the verification analyses, we found that the different dALFF variabilities between the two groups with different window lengths [30 TRs [60 s] and 100 TRs [200 s]] were similar to those of the main findings. In the 30 TRs window length step analyses, the DR group had significantly increased dALFF values in the R-BS, L-CER_10, R-CER_10, L-PHG, R-CER_8, and L-ITG, and decreased dALFF values in the L-MOG and R-MOG relative to the HC group (Supplementary Figure 1 and Supplementary Table 1). Furthermore, in the 100 TRs window length step analyses, the DR group had increased dALFF values in the L-BS, R-BS, L-CER_8, L-CER_4_5, Vermis_6, and decreased increased dALFF values in the L-ITG relative to HC group (Supplementary Figure 2 and Supplementary Table 1).

## Discussion

Our results showed that DR patients exhibited decreased dALFF values in the left middle occipital gyrus and right middle occipital gyrus. Furthermore, DR patients exhibited increased dALFF variability in the right brainstem, left cerebellum_8, left cerebellum_9, and left parahippocampal gyrus.

In our study, we demonstrated that the DR group showed significantly decreased dALFF values in the middle occipital gyrus, which plays an important role in visual information processing. DR patients exhibited progression of retinal hemorrhage and retinal exudate, followed by retinal neovascularization and retinal detachment, which led to vision loss. Thus, reduced retinal input might lead to decreased dALFF values in the visual cortex in DR patients. Moreover, Ozsoy et al. (31) demonstrated that DR patients had decreased N-acetyl-aspartate (NAA) in the visual cortex, accompanied by high HbA1c levels. Another study revealed that DR patients had reduced gray matter density in the right occipital lobe (32). Our previous study demonstrated that DR patients had decreased functional connectivity in the visual network (33). Consistent with these findings, the present study revealed that patients with DR had significantly lower dALFF values in the middle occipital gyrus. dALFF reflects flexibility in spontaneous neural activity, which represents temporal changes in energy consumption and reflects neural network adaptability. Thus, the decreased dALFF values in the middle occipital gyrus might reflect impaired visual processing in DR patients.

In addition, we demonstrated that DR patients exhibited increased dALFF values in the left cerebellum_8 and left cerebellum_9. The cerebellum plays an important role in sensorimotor and vestibular control; it is also involved in cognition and emotion. Heikkilä et al. (34) demonstrated that diabetes does not alter glucose content or uptake in the cerebellum. Fang et al. (35) reported that T2DM patients showed abnormal anatomical connections in the cerebellum. Mazaika et al. (36) found that children with diabetes showed decreased white matter volume throughout the cortex and cerebellum. ÖzdemIr et al. (37) also revealed significant ultrastructural alterations in the diabetic rat cerebellum. In the context of these previous findings, we found that DR patients had significantly increased dALFF in the left cerebellum_8 and left cerebellum_9, which suggests increased flexibility of the brain's activity in the cerebellum. Thus, we speculated that increased flexibility of dALFF in the cerebellum might reflect motor control impairment in DR patients.

Notably, we found that DR patients displayed increased dALFF variability in the left parahippocampal gyrus, which plays an important role in memory (38) and cognition (39). Yau et al. (40) demonstrated that T2DM patients with verbal memory impairment showed abnormal microstructural integrity in the left parahippocampal gyrus. Furthermore, Northam et al. (41) reported that patients with type 1 diabetes exhibited decreased gray matter in the right parahippocampal gyrus and decreased white matter in bilateral parahippocampi, relative to HCs. Grillo et al. (42) also demonstrated that hippocampal insulin resistance was associated with cognitive deficits; thus, the restoration of insulin activity in the hippocampus may be an effective strategy to reduce the cognitive decline in T2DM patients (43). Consistent with these findings, our result revealed that patients with DR had significantly increased dALFF in the left parahippocampal gyrus, which might reflect increased flexibility of brain activity in the left parahippocampal gyrus. Thus, we presume that increased dALFF in the left parahippocampal gyrus might indicate memory and cognitive impairment in DR patients.

Importantly, there were significant differences in the temporal properties of dALFF states between the two groups. Our results revealed that patients with DR showed fewer transitions between states than HCs. Meanwhile, patients with DR showed different mean dwell times in three states, relative to HCs. However, these findings have not been mentioned in previous studies. A previous study demonstrated that patients with Parkinson's disease showed an extended mean dwell time in the segregated state and a reduced number of transitions between states (44). However, the specific neural mechanism underlying these temporal properties remains unknown. Thus, we speculate that these temporal properties of dALFF states constitute potential biomarkers of cognitive impairment in DR patients.

There were some limitations in this study. First, we selected 50 TR as the window length based on the criterion that the minimum length should be >1/*f* min. Moreover, our findings regarding dALFF were relatively stable. Second, our study used a relatively small sample size. We intend to use a larger sample size in future studies.

Our study highlighted that DR patients showed abnormal dALFF in the visual cortices, cerebellum, and parahippocampal gyrus, which might reflect impaired visual, motor, and memory function in DR individuals.

## Data Availability Statement

The raw data supporting the conclusions of this article will be made available by the authors, without undue reservation.

## Ethics Statement

The studies involving human participants were reviewed and approved by Declaration of Helsinki and was approved by the medical ethics committee of the Renmin Hospital of Wuhan University. The patients/participants provided their written informed consent to participate in this study.

## Author Contributions

XH, YS, and ZW contributed to data collection, statistical analyses, and wrote the manuscript. YS designed the protocol and reviewed and edited the manuscript. YS, XH, YT, and C-XQ designed the protocol and contributed to the MRI analysis. YT and C-XQ designed the study, oversaw all clinical aspects of study conduct, and manuscript preparation. All authors contributed to the article and approved the submitted version.

## Conflict of Interest

The authors declare that the research was conducted in the absence of any commercial or financial relationships that could be construed as a potential conflict of interest.
