# Supplementary material for: Dynamic Changes of Amplitude of Low-Frequency Fluctuations in Patients With Diabetic Retinopathy
Source: Front Neurol. 2021 Feb 11;12:611702. doi: 10.3389/fneur.2021.611702 (PMC7905082; doi:10.3389/fneur.2021.611702)
Supplement: Supplementary file 1 [file Table_1.DOCX]

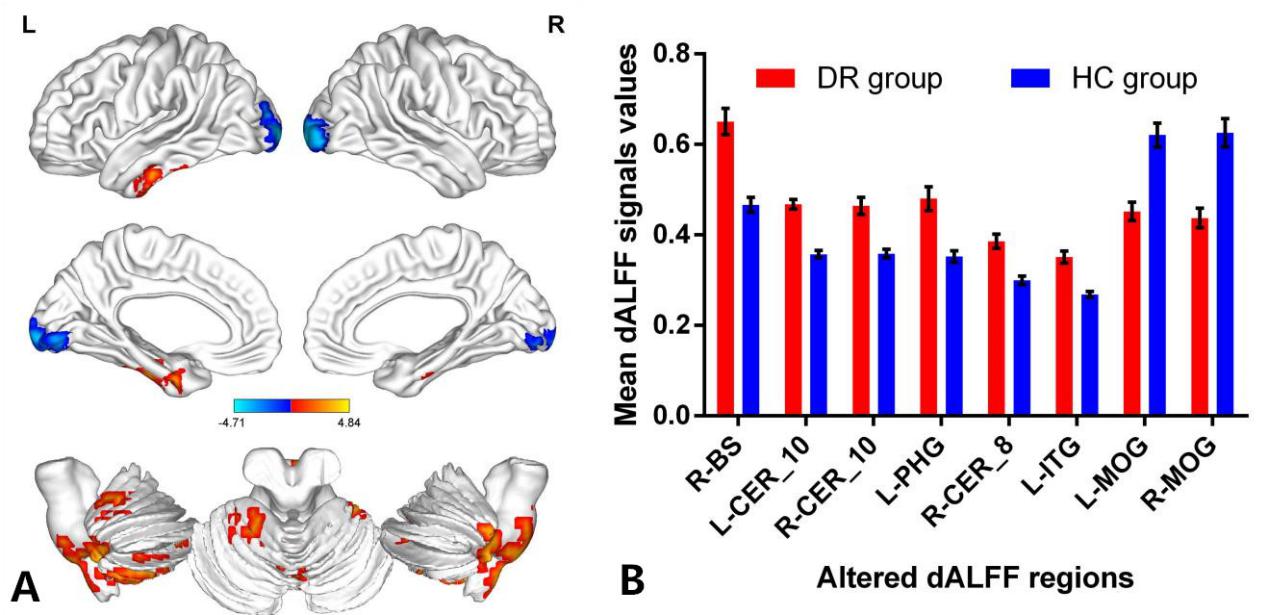


**Figure S1:** Comparison of different dALFF values between DR group and HC group [a window size of 30 TRs (60s), and window shifted by 10 TRs(20s)] .

**Note:**Significant dALFF values differences were observed in the R-BS, L-CER_10,R-CER_10,L-PHG,R-CER_8, L-ITG, L-MOG and R-MOG.The blue areas indicate lower dALFF values.(voxel-level P<0.01, GRF correction, cluster-level P<0.05) (**A**) The mean values **of altered d**ALFF **values between the** DR **and HC groups.**(**B**)

**Abbreviations:** dALFF, dynamic amplitude of low-frequency fluctuation; DR, Diabetic Retinopathy; HC, Health Controls; GRF, Gaussian random field; BS, Brainstem; CER,Cerebelum; PHG, ParaHippocampal; MOG, middle occipital gyrus; ITG, inferior temporal gyrus; L, left; R, right;

**
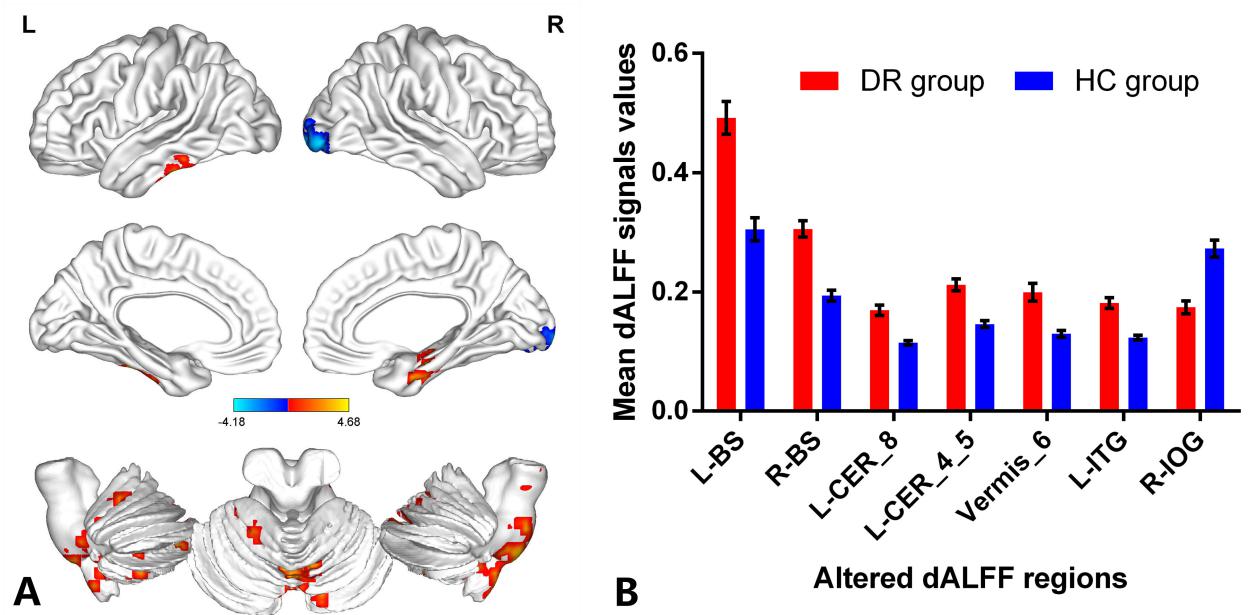
**

**Figure S2:** Comparison of different dALFF values between DR group and HC group [a window size of 100 TRs (200s), and window shifted by 10 TRs(20s)] .

**Note:** Significant dALFF values differences were observed in the L-BS, R-BS,L-CER_8,L-CER_4_5,Vermis_6, L-ITG and R-IOG. The blue areas indicate lower dALFF values.(voxel-level P<0.01, GRF correction, cluster-level P<0.05) (**A**) The mean values **of altered d**ALFF **values between the** DR **and HC groups.**(**B**)

**Abbreviations:** dALFF, dynamic amplitude of low-frequency fluctuation; DR, Diabetic Retinopathy; HC, Health Controls; GRF, Gaussian random field; BS, Brainstem; CER,Cerebelum; IOG, Inferior Occipital Gyrus; ITG, inferior temporal gyrus; L, left; R, right;

**Table S1.** Significant differences in the dALFF between two groups

| Condition/Brain regions | | BA | Peak  T-scores | MNI coordinates | | | Cluster size (voxels) |
| --- | --- | --- | --- | --- | --- | --- | --- |
|  |  |  |  | x | y | z |  |
| [a window size of 30TRs (60s), and window shifted by 10 TRs(20s)] | | | | | | | |
| DR>HC | R-BS | - | 3.9476 | 3 | -33 | -54 | 149 |
| DR>HC | L-CER_10 | - | 4.837 | -18 | -36 | -48 | 414 |
| DR>HC | R-CER_10 | - | 3.8456 | 24 | -36 | -45 | 42 |
| DR>HC | L-PHG, | 28 | 4.0373 | -27 | -3 | -33 | 56 |
| DR>HC | R-CER_8, | - | 3.8992 | 15 | -66 | -39 | 128 |
| DR>HC | L-ITG, | - | 4.2308 | -48 | -30 | -33 | 149 |
| DR<HC | L-MOG | 18 | -4.7133 | -24 | -99 | 3 | 258 |
| DR<HC | R-MOG | 18 | -4.2013 | 18 | -99 | 0 | 200 |
| [a window size of 100TRs (200s), and window shifted by 10 TRs(20s)] | | | | | | | |
| DR>HC | L-BS | - | 4.4355 | -9 | -24 | -48 | 43 |
| DR>HC | R-BS | - | 4.1575 | 9 | -21 | -42 | 94 |
| DR>HC | L-CER_8 | - | 4.6836 | -12 | -63 | -36 | 76 |
| DR>HC | L-CER_4_5 | - | 4.008 | -18 | -39 | -30 | 33 |
| DR>HC | Vermis_6 | - | 3.7761 | 3 | -66 | -27 | 35 |
| DR>HC | L-ITG | 20 | 4.2329 | -54 | -33 | -21 | 30 |
| DR<HC | R-IOG | 17 | -4.1807 | 18 | -96 | -3 | 93 |

**Note:**The statistical threshold was set at the voxel level with p<0.01 for multiple comparisons using Gaussian random-field theory.(voxel-level P<0.01, GRF correction, cluster-level P<0.05).

**Abbreviations:** dALFF, dynamic amplitude of low-frequency fluctuation; DR, Diabetic Retinopathy; HC, Health Controls; GRF, Gaussian random field; BS, Brainstem; CER,Cerebelum; PHG, ParaHippocampal; MOG, middle occipital gyrus; ITG, inferior temporal gyrus; IOG, Inferior Occipital Gyrus; L, left; R, right; B,bilateral;
